# Elements of Decay in American Civilization

**Published:** 1909-07

**Authors:** F. E. Daniel

**Affiliations:** Austin, Texas


### TEXAS MEDICAL JOURNAL.

Established July, 1885.

F. E. DANIEL, M. D., - - - - Editor, Publisher and Proprietor

PUBLISHED MONTHLY.—SUBSCRIPTION $1.00 A YEAR.

VOL. XXV. AUSTIN, JULY, 1909. No. 1.

The publisher is not responsible for the views of contributors.

### Original Articles.

### Elements of Decay in American Civilization.

BY F. E. DANIEL, M. D.,* AUSTIN, TEXAS.

"The preservation of national vigor should be a matter of patriotism." —President Roosevelt.

Everything conceivable, organic and inorganic, in the great universe of God is evolved from a starting point. From the tiniest seed to the magnificent flower with its dazzling tints and its breath of intoxicating perfume; from the embryonic cell, the germ plasm of the human being, to the finished product—moral and intellectual man; from nebulous masses of cosmic matter to blazing suns, there is birth, growth, equilibrium, decline, decay, death in everything; individuals, *races,* nations, worlds, systems and the universe. Look into the heavens and observe in the great nebulæ of Orion and Andromeda the process of sun-building always going on; the birth of new worlds from the germs of extinct worlds that have finished the cycle of evolution. They were born, grew, developed their maximum of life, declined, decayed, died; and their elements are entering into the construction of new worlds, perhaps of a higher order. It is the same with the individual man, and with races and nations. From the parent cell of ancestors, the individual man is evolved, attains his adult existence, develops the best

*Editor TEXAS MEDICAL JOURNAL; late President American International Congress on Tuberculosis, New York, November, 1906; late President Texas State Medical Association; member Texas Academy of Science; member of American Health League and of its "Authors' League"; author of "The Strange Case of Dr. Bruno," etc.

that is in him (perhaps), decays, and passes away, but lives again in his descendants, and thus the cycle of individual existence goes on. Aggregations of individual units make the family, the tribe, the race, the nation. As in the individual, there are in the race elements of decay, which, like the worm in the bud, sap the strength and cause death and decay sooner or later. Nations rise, grow, flourish, decline and pass away, to be followed by others of a higher order (?), and thus is the cycle of evolution perpetuated.

Civilized man in the pride and boast of his intellectuality can conquer these elements, and, to some extent, he does so, and, by their elimination, prolongs human life; e. g., less than a century ago the average length of man's life was about thirty-three years. By the intelligent application of the principles of sanitary science the span of life has been increased to 48 per cent plus. Enough has been accomplished to demonstrate that science can still further lengthen life; life of the individual, the race, the nation, by the further removal of the elements of decay.

In a brief magazine article I can only allude to the conquest of smallpox, yellow fever, diphtheria, cholera and bubonic plague to show to what extent science can benefit man, if government will heed its voice. For a masterly account of the achievements of sanitation in other countries, readers are referred to an article by Surgeon Major (U. S. A. retired) L. L. Seaman, New York, in the *New York Medical Journal*, February 22, 1908.

It behooves us, then, to carefully consider what are those elements of decay which threaten our national existence, for, in my deliberate opinion, we are pursuing, through neglect of causes, a course that can only be denominated race suicide; and to inquire in how far it is possible to remove them. The history of the world shows a singular unanimity of causes and effects in the rise, progress, decline and death of civilization. Rome may be taken as the type. America seems to be following in her footsteps, and it can be shown that the immediate and remote causes that led to the decline and fall of the one are operating to insure the decline and fall of the other. Will rational man not profit by experience? The ability to do so is held to be the principal argument to prove man's superiority in the animal kingdom. After seven hundred years of uninterrupted conquest Rome was mistress of the world. Surfeited with power and glory, inordinate wealth, the spoils of Europe, Asia and Africa, luxury, debauchery, idleness, drunkenness and disease,—the neglect of the right principles of living, she made her first mistake in welcoming to her territory vast hordes of barbarians from beyond the Danube—"undesirable citizens." She gave them land, and by a process of "benevolent assimilation" absorbed and interbred with them, and finally hired them to do their fighting. The martial spirit that had conquered the world (Emmerton, Gibbon) was dead. It was the story of the camel that got his head in the stable door. Soon Atilla, Alaric and Genseric did the rest,—and where is Rome today? A memory only. The United States government made the initial mistake in permitting the importation of black cannibals as slaves,—from whom have descended ten millions of the most undesirable citizens; and our people have mixed with them until, according to Surgeon Major R. W. Shufeldt (U. S. A., retired), in his wonderful book, "The Negro, a Menace to American Civilization," fully one-half of them have an admixture of white blood; and, although intermarriage with them is prohibited in most States, miscegenation is going on, with all the evils to our race attendant upon hybridization,—the admixture with inferior blood. Major Shufeldt says that if this goes on, in course of time, no man, woman or child in America can be sure that he has no negro blood in his veins.

The United States government made the further mistake of welcoming to its shores and absorbing countless hordes of individuals of inferior races—the scourings and outcasts of the world; not the true immigrants so needed and so cordially welcomed in the young days of our Republic, but paupers, syphilitics, consumptives, criminals, anarchists. The American people have already lost most of their race characteristics by interbreeding with these, until we are rapidly approaching, if we have not become, a race of mongrels. Some of these people were paupers on arrival, but amassed great wealth, and became the ancestors of some of our "aristocracy." And their descendants, drunk with wealth and luxury, have ceased to breed, or, if they have offspring, it is fewer and feebler by reason of the effeminacy induced by luxury, idleness and dissipation. The tendency here is to race suicide.

Francis Amasa Walker says: "* * * For nearly two generations great numbers of persons utterly unable to earn their living, by reason of one or another form of physical or mental disability, and others who were, from widely different causes, unfit to be members of any decent community, were admitted to our ports without challenge or question. It is a matter of official record that in many cases these persons have been directly shipped to us by states or municipalities desiring to rid themselves of a burden and a nuisance; while it could reasonably be believed that the proportion of such instances was far greater than could be officially ascertained. * * *" But he continues, "The question today is not of preventing the wards of our almshouses, our insane asylums and our jails from being stuffed to repletion by new arrivals from Europe, but of protecting the American rate of wages, the American standard of living, and the *quality of American citizenship* from degradation through the tumultuous access of vast throngs of ignorant and brutalized peasantry from the countries of eastern and southern Europe." (*Economics and Statistics*, Vol. II, pp. 437-449, Henry Holt & Co., New York, 1899.)

Of this class, doubtless, came the Orchards, the Harry Thaws, the Czolgozs, Prendergasts, the Guiteaus, *et id. om. gen.* It is with this class and their undesirable progeny that we have to deal today, and for whom we must provide asylum and jail, electric chairs, gallows and poorhouses. This is as irrational as trying to disinfect a sewer at the mouth, while the current of filth flowing into it is ever increasing. Mr. Strauss has a task before him as futile as that of the Danaides, or of Sisyphus, who perpetually rolled the stone. And he may as well whistle to the wind!

Macaulay (*Letters and Addresses*), in 1857, writing to Henry S. Randall, the Speaker of the House of Representatives in Congress, said: "I have long been convinced that institutions, purely democratic, must, sooner or later, destroy liberty or civilization or both. Your Constitution is all sail and no anchor. Either some Cæsar or Napoleon will seize the reins of government with a strong hand, or your Republic will be as fearfully plundered and laid waste by barbarians in the twentieth century as the Roman Empire was in the fifth, with this difference, that the Huns and Vandals, who ravaged the Roman Empire, came from without, and that your Huns and Vandals will have been engendered within your own country by your own institutions."

How much longer will rational man attempt to correct *results* and ignore the causes,—causes that should never have existed,—and some of which,—many of which,—can even now be removed and should be removed in the interest of humanity and race integrity? It is too late now to remedy the evils that have followed the introduction of the black cannibals and of the criminals and paupers into the country, unless we consider Major Shufeldt's suggestion to segregate the blacks and possibly avoid further evil,—possibly a greater one of race war; and Mr. Strauss is now striving to separate and deport the imported anarchists and criminals. The mischief is done.

But there are elements of race degeneracy now at work and which, although their influences are far-reaching and destructive, by wise legislation, based on a decent regard by Congress for the voice of science, may yet be eliminated,—at least, greatly modified. They are well within the scope and power of sanitary science, a science which aims at the cause and its removal, as in the cases cited,—the conquest of epidemic diseases. Excepting, therefore, the causes of race degeneracy above mentioned as the chief factors, and which are beyond sanitary science, I name as the most potent factor in the swift decay of the race,—the worm in the bud gnawing at its vitals,—whisky and consumption. In his inaugural address as President of the American International Congress on Tuberculosis, held in New York, November 15, 1906, the writer said:

*"Whisky and consumption follow the Bible and the flag as the handmaids of civilization, and they are as destructive as a pestilence."*

This was flashed around the world and appeared in thousands of papers under sensational headlines, as if it were some new truth just discovered, when it is patent to every student of history or of social or political economics.

The statistics of consumption have been given so often that to a medical man it seems trite and threadbare. But to many who read this it will, perhaps, be startling news that, while the mortality of yellow fever for one hundred years (U. S. census) averages one thousand a *year*, consumption now destroys one thousand lives every *two and a half days!* And yet, so far as I am aware, the government raises not a hand to stay its ravages. No fact is better established than that the disease is not hereditary, as is insanity, for instance, and of which I will speak presently; and that its spread from the sick to the well can be prevented, if government would prevent it. Suppose four hundred people were being slain every day,—night and day, year in and year out,—by some armed invasion, for instance, or one one-hundred and fiftieth as many (for that is the relation of the mortality of yellow fever to that of consumption), what would be the state of public sentiment and the attitude of Congress on the subject? Every resource of science and power would be brought into action to arrest it, and yet consumption destroys 150,000 lives annually.

I have not space to speak of the sick who finally die, the money loss to the country by reason thereof, the poverty, want, suffering, *prostitution,* the enfeebled and fewer progeny of consumptive parents. Nor can I enter into a consideration of certain other destructive factors, however far-reacing and ruinous; e. g., venereal disease, increase of crime, and waste of natural resources. The latter belong to another department of science. Nor of child-labor,—"grinding our seed corn," Lincoln said. These all tend to race extinction.

In the nearly three score and ten years of the writer's life, the race has dwindled physically it seems to him; the men are smaller and feebler. A strong, broad-shouldered, manly man is the exception. For the most part, the rising generation of young men within my observation are small, narrow-chested, stoop-shouldered, slim-legged little shrimps, and they are to be the fathers of the next generation! What will their children be? Their inheritance will be neurasthenia, unstable constitution, mayhaps the curse of alcoholism or of alcoholic insanity.

Now as to alcohol, the cause, and insanity, crime, pauperism, prostitution, sin, sorrow and human misery, the result.

The population of Texas in forty-five years to 1904 increased 504 per cent. The insane in Texas in the same time increased 6800 per cent, a ratio of 13.7 to 1. In 1860 there were fifty insane people in Texas, and their maintenance cost the State $12,000. These figures are from a public and published address by Prof. M. L. Graves, M. D., University of Texas, late Superintendent Southwestern Insane Asylum, San Antonio, Texas.

In 1908 there are 5000 insane in Texas (500 in jail for lack of asylum accommodation), and the cost to the State for the five years to 1904 was $3,555,000, an average of $707,000 a year. For 1904 alone the cost was $784,000. These figures are taken from the Comptroller's books.

According to Dr. Graves, 60 per cent of the insane in Texas is the result of alcohol, directly and by hereditary transmission; while Dr. B. M. Worsham, Superintendent of the Austin (Texas) Insane Asylum, puts it at 95 per cent. Further along, I give official figures from superintendents of twenty-two asylums in sixteen States. The entire revenue to Texas from liquor licenses, according to Judge T. W. Gregory, of Austin, in a public address, is $600,000. It will be seen that this large sum falls short by an average of $107,000 a year of paying for taking care of the insane, from 60 per cent to 95 per cent of whom are, according to official reports of the superintendents of two of our four asylums, victims of drink and hereditary transmission of the curse; while Judge Gregory declares that the cost of enforcing the criminal laws in Texas exceeds the entire revenue from liquor license, and that fully 50 per cent of crime is the result of drink.

The figures are astounding. To continue a policy the results of which are so destructive to the human family, so fatal to our race integrity, is race suicide! Nay, it is idiotic! We let these people marry and multiply. The insane, the criminal, the consumptive, the scorbutic, the syphilitic, aye, even the paupers on the poor farm, marry with the sanction of the law and the blessing of the church! That is sentiment. Science would insist upon stirpiculture—the rational propagation of the human species—else the fittest can not survive. Lydston, in his recent epoch-making book, "Diseases of Society," says that a feeble dude with a monocle in his eye gazed through the bars of a pasture upon the splendid proportions of a magnificent bull, and, addressing the bull, said: "By Jove, old fellow, you are a splendid specimen of your kind, don't you know?" The bull replied: "I'm sorry, my son, that I can't say the same for you; you are a miserable specimen of yours, don't you know? But I'll give you a pointer: If the same pains and care had been taken in the selection of your ancestors that were taken with mine, you would not have been the miserable little failure you are, don't you know?" or words to that effect.

Under nature's laws, unhampered by man's mistaken sentiment of humanity (humanity to the individual, inhumanity to the race), the fittest survive. All our humanitarian work, pity for the defectives, fosters the survival of the unfit by the perpetual propagation of the defectives, which, left to the operation of natural law, would be eliminated. Tennyson says of Nature: "So careful of the type she seems, so careless of the single life." This formula may be paraphrased, and made to express an unfortunate truth when we say, "Man, so careful of the defective life, so reckless of the race." Darwin says the families of drunkards do not descend beyond the fourth generation, they die out,—thus verifying the Scripture as to the sins of the father, etc. But the United States government and State governments, *encouraging, promoting and facilitating drunkenness,* by putting a tax on alcohol for revenue, and licensing the indiscriminate sale by retail, of liquor, most frequently poisoned by adulteration to cheapen it, must feel some obligation to take care of its victims, and they endeavor to do so, by expending an amount for lunatic asylum accommodations alone greatly in excess of the revenue, and another amount equally in excess of revenue for the prosecution and punishment of another class of its victims—the criminal. Is the game worth the candle?

The following is summarized from reports of asylum superintendents in the States named, and furnished the TEXAS MEDICAL JOURNAL (August, 1907), by Dr. C. H. Gregory, Superintendent of the North Texas Insane Asylum, Terrell, Texas:

Illinois, Watertown, 30 per cent of insanity due to alcohol.

Michigan, Newberry, 32 per cent.

Missouri, St. Louis, 55.5 per cent.

New York, Utica, 11.28 per cent (in men alone, 19.3 per cent).

New York, Rochester, 48 per cent (10 per cent women).

Massachusetts, Hawthorn, 18 to 20 per cent.

Massachusetts, Fredericksburg, 25 per cent.

Massachusetts, Harding, 27 per cent.

Massachusetts, Worcester, 33.3 per cent.

Vermont, Brattleboro, 35 per cent.

Pennsylvania, Morristown, 20 to 25 per cent.

Pennsylvania, Warren, 25 per cent.

New Hampshire, Concord, 35 per cent direct, 40 per cent hereditary alcoholic.

Nebraska, Inglesides, 30 plus per cent.

California, Stockton, 16 per cent.

Washington, Medicine Lake, 40 per cent.

West Virginia, Spencer, 15 per cent.

Kansas, Topeka, 35 per cent.

Colorado, Pueblo, 42 per cent (7 per cent in females).

Wisconsin, Wauwatosa, 16 per cent.

Texas, Austin, 95 per cent.

Texas, San Antonio, 60 per cent.

Twenty-two asylums in sixteen States.

Average each State, 46.75 per cent.

Average each asylum, 34 per cent.

Tabulated in the order of their importance, the curable and the incurable elements of decay in our civilization, I restate the proposition about like this:

Miscegenation with the descendants of black savages only a few generations removed from cannibal ancestors.

Interbreeding with the refuse of Europe,—the criminal, the anarchist, the pauper.

Licensing the indiscriminate sale of an agent which statistics show to be the most destructive of all agents to life, health and morals,—the chief factor in the production of insanity, crime, pauperism, prostitution, disease, death and human misery.

Child-labor. Increase of crime. Waste of natural resources.

Our criminally lax marriage laws, which encourage the breeding of defectives.

These are beyond the reach of sanitary science, and belong to that of economics.

Consumption,—often the indirect result of intemperance,—and other preventable diseases, which, it is estimated, carry off four-fifths of all who die prematurely.

Venereal diseases.

What sanitary science has done in other countries it can do in this. And this is the task to which the Committee of One Hundred on Federal Legislation, in collaboration with the Association for the Advancement of Science now addresses itself. While we may never be "civilized" up to a scientific breeding of people (as we do our stock), or totally eradicate disease, it unquestionably lies within the power and scope of science to eliminate much that is evil, and bring about great improvement, even in the next generation, in the physical, moral and intellectual status of society. To this end government should create a Department of Public Health, officered by the ablest men, and beyond the reach of politics, and make an appropriation adequate to meet the requirements, however expensive it may be. It will be true economy in the end. This end,—the elimination of the preventable and the cure of the curable elements of race decay,—is the mission of rational medicine, to which all "exceptional men," the enlightened, should address themselves. Duty requires it; true philanthropy dictates it; policy suggests it, and it is demanded by every consideration of humanity and race integrity. Will Congress heed the warning?

Austin, Texas, 1908.

